# A Unique Evolution of the *S2* Gene of Equine Infectious Anemia Virus in Hosts Correlated with Particular Infection Statuses

**DOI:** 10.3390/v6114265

**Published:** 2014-11-10

**Authors:** Xue-Feng Wang, Shuai Wang, Qiang Liu, Yue-Zhi Lin, Cheng Du, Yan-Dong Tang, Lei Na, Xiaojun Wang, Jian-Hua Zhou

**Affiliations:** 1 State Key Laboratory of Veterinary Biotechnology, Harbin Veterinary Research Institute of Chinese Academy of Agricultural Sciences, Harbin 150001, China; E-Mails: xuefengwang1982@126.com (X.-F.W.); 8111781252@163.com (S.W.); liuqiangtriumph@163.com (Q.L.); sndhr@163.com (Y.-Z.L.); chengdu1981@163.com (C.D.); tangyandong2008@163.com (Y.-D.T.); nl2zy@163.com (L.N.); 2 Harbin Pharmaceutical Group Biovaccine Company, Harbin 150069, China

**Keywords:** equine infectious anemia virus, EIAV, *S2* gene, evolution, diversity

## Abstract

Equine infectious anemia virus (EIAV) is a member of the *Lentivirus* genus in the *Retroviridae* family that exhibits a genomic structure similar to that of HIV-1. The S2 accessory proteins play important roles in viral replication *in vivo* and in viral pathogenicity; however, studies on *S2* evolution *in vivo* are limited. This study analyzed the evolutionary characteristics of the *S2* gene of a pathogenic EIAV strain, EIAV_LN40_, in four experimentally infected horses. The results demonstrated that 14.7% (10 of 68 residues) of the stable amino acid mutations occurred longitudinally in S2 during a 150-day infection period. Further analysis revealed that six of the ten mutated residues were positively selected during the infection. Alignment and phylogenetic analyses showed that the *S2* gene sequences of viruses isolated from the infected horses at the early stage of EIAV_LN40_ infection were highly homologous and similar to the vaccine-specific sequence. The *S2* gene variants isolated from the febrile episodes and late phase of infection became homologous to the *S2* gene sequence of the inoculating EIAV_LN40_ strain. Our results indicate that the *S2* gene evolves in diversity and divergence *in vivo* in different stages of EIAV infection and that this evolution correlates with the pathogenicity of the virus.

## 1. Introduction

Equine infectious anemia virus (EIAV) belongs to the *Lentivirus* genus in the *Retroviridae* family. This virus predominantly infects *Equidae*, and the onset of disease typically occurs within three to four weeks post-infection. Viral replication could be controlled in most infected horses after approximately one year of infection, and these horses become asymptomatic carriers for life. Carriers can transmit the virus through blood, and the clinical symptoms of equine infectious anemia (EIA) might recur in carriers when the immune system is naturally or experimentally suppressed [1]. These phenomena indicate that infected horses can control EIAV replication without eliminating the virus. Therefore, EIAV has been recognized as a unique model for the study of *lentivirus* replication under immune control [2].

The EIAV genome is the least complex among lentiviruses. In addition to the structural proteins encoded by the *gag*, *pol*, and *env* genes, EIAV encodes three small proteins, which include the Tat and Rev regulatory proteins and the S2 accessory protein [3]. The *S2* gene encodes a protein of 65 or 68 amino acid residues [4,5]. The S2 protein is unique to EIAV, and its function is unclear. The S2 protein potentially interacts with Gag, without incorporation into EIAV particles. It has been reported that the S2 protein interacts with the cellular protein OS-9 [6]. The S2 protein has no sequence homology with other *lentivirus* proteins. There are a few common predicted motifs that are shared in the amino acid sequences of EIAV S2 and the primate *lentivirus* Nef, including a myristoylation site, an SH3 domain binding motif and a casein kinase 2 phosphorylation site. Therefore, EIAV S2 might have a function similar to Nef of HIV-1 or SIV [7]. The HIV-1 Nef protein is an important factor associated with the virulence and infectious efficacy of the virus. Studies have demonstrated that the primate lentivirus *Nef* gene has numerous polymorphisms and presents different variants in different disease states *in vivo* [8,9].

The EIAV *S2* gene showed remarkable variation after successive passages in donkey monocyte-derived macrophages (dMDM), an attenuating process that greatly reduced the pathogenicity of the precursor EIAV_LN40_ [5]. Deletion of *S2* in EIAV does not affect viral replication *in vitro* [10]. Additionally, the information regarding *S2* evolution in infected horses is limited. Two studies that used the EIAV_PV_ infectious clone-derived virus to infect ponies found that the EIAV *S2* gene was highly conserved in infected animals *in vivo* [11]. The deletion of *S2* significantly reduced viral replication *in vivo* and attenuated the virulence of the virus [11]. Our previous studies on the Chinese EIAV attenuated vaccine revealed that the *S2* gene was one of most varied regions compared with its parental virulent strain [5]. The reversal of four major stable mutations in the *S2* gene of an infectious clone of the vaccine EIAV strain (EIAV_FDDV3-8_) in the sequence of the virulent strain resulted in a modest but statistically significant increase in the plasma viral load and clinical signs (increased body temperature and decreased platelet number) [12]. This result suggests that the S2 protein is crucial; however, it is not the only factor affecting EIAV pathogenicity *in vivo*. In this study, we longitudinally analyzed the sequence of the *S2* gene during the first five months of EIAV_LN40_ infection in horses. The results demonstrated that the *S2* gene is highly diverse *in vivo* and significantly evolves under positive selection pressure.

## 2. Materials and Methods

### 2.1. EIAV Strains

EIAV_LN40_ is a virulent strain derived from a field strain isolated in Liaoning Province in China by 16 successive passages in horses. EIAV_LN40_ is highly lethal in horses; however, it causes no apparent clinical EIA symptoms in donkeys. An attenuated, live EIAV vaccine, EIAV_DLV121_, was developed by passaging EIAV_LN40_ in donkey monocyte-derived macrophages (dMDMs). A fibroblast-adapted derivate, EIAV_FDDV13_, was subsequently developed by 13 passages of EIAV_DLV121_ in fetal donkey dermal (FDD) cells. EIAV_DLV121_ and EIAV_FDDV13_ proliferate in horses and donkeys without causing EIA symptoms and induce protective immunity against experimental and natural infections with pathogenic EIAV strains. These three EIAV strains were stocked at the Harbin Veterinary Research Institute of the Chinese Academy of Agricultural Sciences [5].

### 2.2. Horses Experimentally Infected with EIAV

Four horses were infected with EIAV in a previous study [13]. Briefly, two male 4-year-old horses (#25 and #26) were subcutaneously injected in the neck with 1 × 10^6^ TCID_50_ of EIAV_LN40_; these animals died from typical EIA at day 28 and day 30 days post infection (dpi), respectively. Another two male 4-year-old horses (#4 and #10) were subcutaneously injected in the neck with 10-fold less EIAV_LN40_ (1 × 10^5^ TCID_50_). No EIA clinical symptoms were observed for 5 months post infection in both horses. Samples of 100 ml of peripheral blood were taken at the time points indicated in Figure S1.

The use of horses and the related experimental protocols in this study were approved by the Institutional Animal Care and Use Committee (IACUC) of the Harbin Veterinary Research Institute (HVRI), Chinese Academy of Agricultural Sciences. At the end of the experiment, or when severe disease-associated symptoms resulting in distress appeared, the horses infected with pathogenic EIAV strains were euthanized by an intravenous injection of pelltobarbitalum natricum (100 mg/kg body weight, dissolved in saline) in the jugular vein by veterinarians according to protocols approved by the IACUC of HVRI.

### 2.3. Analysis of S2 Gene Variation

Virions were collected from the plasma of the blood samples from the horses infected with EIAV_LN40_ or culture supernatants of equine monocyte-derived macrophages (eMDMs) infected with EIAV_DLV121_ and fetal donkey dermal (FDD) cells infected with EIVA_FDDV13_ by centrifugation. The viral genomic RNA was extracted from the pellets using a QIAamp Viral RNA Mini Kit (QIAGEN, Hilden, Germany). The full-length *S2* gene cDNA fragments were amplified by three independent nested reverse transcription (RT)-PCR experiments, as previously described [13]. The PCR products were excised from 0.8% agarose gels and ligated into the pMD18-T vector (TaKaRa, Dalian, China). Eight to 25 positive recombinant clones of each sample were sequenced. The alignment and phylogenetic analysis of the nucleotide sequences were performed with the SeqMan II tool of the Lasergene DNAStar program (version 6.0, DNAStar Inc., Madison, WI, USA, 2001) and the Molecular Evolutionary Genetics Analysis (MEGA) program (version 5.0, Center for Evolutionary Functional Genomics Biodesign Institute, Arizona State University , Tempe, AZ, USA, 2011). The phylogenetic tree was constructed with nucleotide sequences using the bootstrap neighbor-joining method. The bootstrap values were calculated from 1000 replicates of the alignment. Statistical analysis of sequence variations in S2 sequences was performed by SAS (Statistical Analysis System) (version 9.2, SAS Institute Inc, SAS Campus Drive, Cary, NC, USA, 2008).

### 2.4. Detection of Selection Pressures

The detection of the selection pressures of the evolutionary processes of the EIAV *S2* gene was performed using the codeml method of the PAML software package (version 4.3, Department of Biology, University College London, London, UK, 2009) [14]. The ratio ω of non-synonymous to synonymous substitutions (d_N_/d_S_) is an important indicator of selection pressure at the codon level, and ω = 1, <1 and >1 reflect neutral, purifying and positive selection, respectively. The Model M0 (ratio of one), M1a (nearly neutral), M2b (positive selection), M3 (discrete), M7 (β) and M8 (β and ω) of the codeml program are typically applied in the detection of codon-specific positive selection in a virus gene codon [14,15,16].

## 3. Results

### 3.1. The S2 Gene Highly Varied among the Isolates of Experimentally Infected Horses and in Vitro Attenuated Strains

Four horses were experimentally infected with a pathogenic EIAV strain, EIAV_LN40_, in a previous study of the evolution of EIAV *in vivo* [13]. Two of these horses (#25 and #26) were inoculated with 1 × 10^6^ TCID_50_ EIAV_LN40_ and died of acute EIA at 28 and 30 days post infection (dpi), respectively. Another two horses (#4 and #10) were infected with 10-fold less of the virus, which resulted in sub-clinical infections (Figure S1). 

To investigate the evolution of the *S2* gene in long-term infection in the host, the *S2* gene fragment was amplified by RT-PCR from 18 plasma samples of four horses infected with EIAV_LN40_. The PCR product was not obtained from Sample 4-1, which indicates Time Point 1 of horse #4. In addition, the *S2* genes of two attenuated EIAV strains, *i.e.*, eMDM-adapted EIAV_DLV121_ and FDD-adapted EIAV_FDDV13_, were amplified from virions collected from the culture supernatants of the infected cells. Eight to 26 *S2* nucleotide sequences were determined from randomly selected PCR clones of each sample, and a total of 387 nucleotide sequences (HQ008940-HQ009261, HQ223289-HQ223335) were obtained. The S2 nucleotide and deduced amino acid sequences of EIAV_LN40_ isolated from experimentally infected horses were compared with the S2 sequence of EIAV_LN40_ before inoculation, as well as those of two attenuated strains, EIAV_DLV121_ and EIAV_FDDV13_.

**Table 1:** Comparison of nucleotide and amino acid genetic distances of the *S2* genes of viruses isolated from different samples infected with EIAV_LN40_ and the vaccine strains EIAV_DLV121_ and EIAV_FDDV13_.

Sample		EIAV_LN40_		EIAV_DLV121_		EIAV_FDDV13_	
name	dpi	nt	aa	nt	aa	nt	aa
DLV121	V	2.32(0.97–4.53)	6.84(2.99–14.20)				
FDDV13	V	2.31(1.47–4.00)	6.65(4.51–10.86)	1.17(0–3.49)	3.21(0–7.64)		
25-1	14	2.75(2.47–4.00)	7.02(6.06–10.86)	1.81(0.49–3.50)	5.22(1.48–10.86)	1.78(0.98–3.50)	4.96(2.99–7.64)
25-2 *	18	0.44(0–1.96)	1.03(0–4.51)	2.32(0.97–4.51)	6.78(2.99–12.52)	2.31(1.47–3.99)	6.60(4.51–9.24)
25-3 *	28	0.32(0–1.47)	0.74(0–4.51)	2.21(0.97–4.00)	6.50(2.99–12.52)	2.20(1.47–3.49)	6.30(4.51–9.24)
26-1	14	2.89(2.47–4.50)	8.57(7.64–14.20)	1.84(0.49–3.47)	5.28(1.48–10.86)	1.79(0.98–3.47)	5.04(2.99–9.24)
26-2 *	18	1.74(0–4.00)	5.17(0–10.86)	1.37(0–3.49)	3.97(0–10.86)	0.73(0–2.97)	1.88(0–7.64)
26-3 *	30	0.35(0–1.47)	0.87(0–4.51)	2.17(0.49–4.00)	6.43(1.48–12.52)	2.15(0.98–3.49)	6.23(2.99–9.2)
4-2	28	2.05(0.98–3.49)	6.2(2.98–10.86)	2.17(0.49–5.04)	6.51(1.48–14.20)	1.81(0.98–3.49)	5.27(2.99–9.24)
4-3	42	1.69(1.47–2.47)	5.03(4.51–7.64)	1.42(0.49–2.97)	4.18(1.48–7.64)	0.64(0.49–1.47)	1.66(1.48–2.9)
4-5	70	2.04(1.47–4.00)	5.72(4.51–10.86)	2.41(0.49–5.59)	6.86(1.48–14.20)	1.94(0.98–4.02)	5.27(2.99–9.24)
4-7	112	2.36(1.97–3.50)	3.92(2.99–9.24)	3.67(1.47–6.11)	7.96(2.99–14.20)	3.2(1.47–4.53)	6.26(2.99–9.2)
4-8	162	1.88(0.98–3.49)	5.47(2.99–10.86)	1.31(0.49–3.47)	3.68(1.48–9.24)	0.90(0-2.48)	2.30(0–6.06)
10-1	14	2.8(2.47–4)	8.44(7.64–12.52)	1.75(0.49–2.99)	5.08(1.48–9.24)	1.70(0.98–2.99)	4.83(2.99–7.64)
10-2	28	2.72(2.47–4)	8.27(7.64–12.52)	1.67(0.49–2.99)	4.90(1.48–9.24)	1.62(0.98–2.99)	4.67(2.99–7.64)
10-3	42	2.34(0.49–3.49)	6.92(1.48–10.86)	2.26(0.49–5.57)	6.59(1.48–14.20)	2.12(0.98–4.00)	6.06(2.99–10.81)
10-5 *	70	0.87(0.49–2.48)	2.07(1.48–4.51)	2.77(1.47–5.06)	7.90(4,51–12.52)	2.76(1.96–4.53)	7.70(6.06–9.24)
10-7	112	0.86(0.49–2.48)	2.38(1.48–6.06)	2.77(1.47–5.06)	8.25(4.51–14.20)	2.76(1.96–4.54)	8.05(6.06–10.81)
10-8	162	1.34(0.97–2.47)	3.85(2.99–7.6)	1.83(0.98–4.00)	5.37(2.99–10.87)	1.28(0.98–2.48)	3.53(2.99–6.06)

EIAV: Equine Infectious Anemia Virus; * Indicates typical febrile episodes; dpi: days post infection; nt: nucleotide; aa: amino acid; V: vaccine.

**Figure 1:**
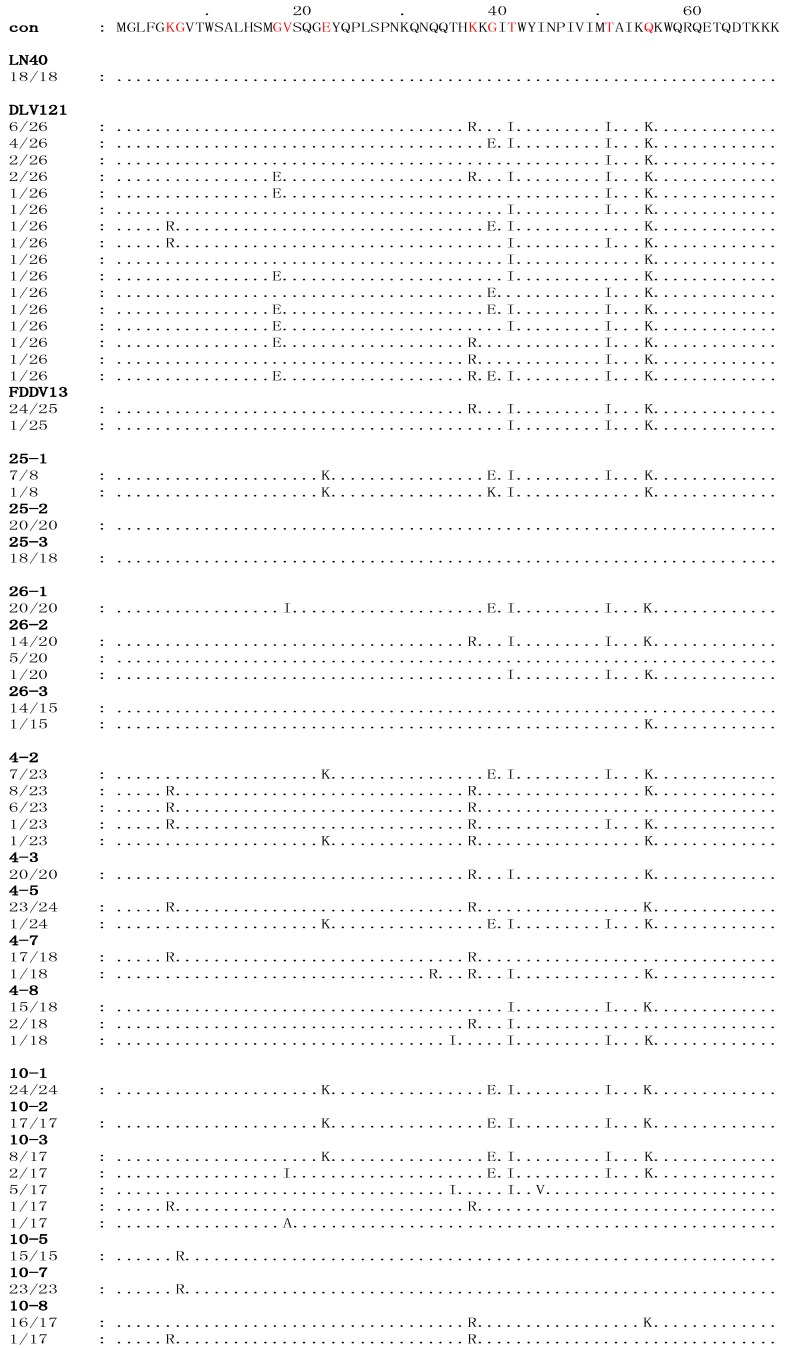
The deduced multiple the deduced sequence alignment of amino acids encoded by the *S2* genes of viruses isolated from four EIAV_LN40_-infected horses. The sequences of all the viral clones were aligned to the consensus sequence (con) of EIAV_LN40_, shown at the top. Only the amino acid residues that are different from the consensus sequence of EIAV_LN40_ are indicated. The dots indicate residues identical to the consensus sequence. The numbers in the left column in each sequence indicate the number of a specific sequence in the total detected clones of each sample. The red letters denote the stable substitutions.

As summarized in Table 1, the divergence of the *S2* nucleotide sequence between EIAV isolated from the four horses after inoculation for 150 days and EIAV_LN40_, EIAV_DLV121_ and EIAV_FDDV13_ were 0%–4.50%, 0%–6.11%, and 0%–4.54%, respectively. The percentages of divergence of the corresponding amino acid sequences were 0%–14.20%, 0–14.20, and 0%–10.81%, respectively. An alignment analysis of the deduced S2 amino acids sequences between the EIAV_LN40_ consensus reference and the isolates from experimentally infected horses was performed (Figure 1). The variable amino acid positions were scored by the existence of at least three clones containing an amino acid residue that differed from the EIAV_LN40_ consensus sequence, which was summarized from 18 clones. The results revealed that ten stable amino acid variations in S2, including 6K/R, 7G/R, 17G/I, 18V/I, 22E/K, 37K/R, 39G/E, 41T/I, 51T/I, and 55Q/K (Figure 1). These mutated residues accounted for 14.7% (10/68) of the total deduced S2 amino acid residues.

### 3.2. Mutations in the S2 Gene Were the Result of Positive Selection

To investigate whether the mutations in *S2* occurred randomly or as a result of evolutionary pressure, which implicates the involvement of the target gene in an altered phenotype, the *S2* sequences were analyzed for the evidence of positive selection using a maximum likelihood (ML) method implemented in the PAML4.3 software package. This method is based on the ratio of non-synonymous (dN) to synonymous (dS) substitutions. Three model pairs (M0/M3, M1a/M2a, and M7/M8) were employed to evaluate the likelihood of positive selection for the mutations in *S2*. The expected ratio (ω) of the dN to dS substitutions in a gene is one (ω = 1). The former models in these pairs are neutral models that do not permit positive selection (ω < 1), and the latter ones are alternative models that permit positive selection (ω > 1). The analytic data shown in Table 2 indicate that the comparisons of M0 to M3, M1a to M2a, and M7 to M8 were significant and that the models that permit positive selection are a better fit to these data, *i.e.*, the *S2* gene was under positive selection pressure. The amino acids that are most likely responsible for the non-neutral pattern were detected. Six codons (17, 18, 22, 37, 39, and 41, see Table 2) appeared to be under positive selection pressure (the posterior probabilities were over 95%).

**Table 2:** Summary of the parameter estimates of different codon evolution models for the EIAV *S2* gene. dN: non-synonymous; dS: synonymous.

Model	dN/dS	Parameters ^b,c^	2ΔlnL	Positively Selected Codons ^d^
M0 (ratio of one) 1 ^a^	2.2197	ω = 2.2197	34.71 *p* < 0.01	None
M3 (discrete) 5 ^a^	2.4099	P_0_ = 0.54824 p_1_ = 0.34821 p_2_ = 0.10355 ω_0_ = 0.79215 ω_1_ = 3.59132 ω_2_ = 7.00158		17, 18, 22, 37, 39, 41
M1a (neutral) 1 ^a^	0.8434	p_0_ = 0.17661 p_1_ = 0.82339 ω1 = 0.11319 ω_1_ = 1.00000	47.544 *p* < 0.01	Not permitted
M2a (selection) 3 ^a^	2.4296	p_0_ = 0.06756 p_1_ = 0.56448 p_2_ = 0.36796 ω_0_ = 0.32811 ω_1_=1.00000 ω_2_ = 5.00850		17, 18, 22, 37, 39, 41
M7 (β) 2 ^a^	1.0000	P = 97.53765 q = 0.00500	51.384 *p* < 0.01	Not permitted
M8 (β & ω) 4 ^a^	2.4337	p_0_ = 0.63252 p_1_ = 0.36748 p = 0.19920 q = 0.01258 ω = 5.02072		17, 18, 22, 37, 39, 41

^a^ Number of degrees of freedom used; ^b^ Values in parentheses are not free parameters; ^c^ Parameters p and q are the shape parameters of the beta distribution that underlies M7 and M8; ^d^ Codons inferred to be under selection at a level of 85%. Numbers in bold refer to codons with posterior probabilities over 95%.

### 3.3. The Vaccine- and Pathogenic-Specific S2 Sequences Were Identified in Inoculated Horses, Depending on the Inoculation Dose and Infection Status

To examine whether the *S2* gene evolved at different infection stages to best fit the environmental and immunity pressures of the host, the mutations that presented as changes in the deduced amino acid residues in the S2 protein were longitudinally analyzed by comparing the *in vivo* isolated sequences with the consensus S2 sequence of EIAV_LN40_.

**Figure 2:**
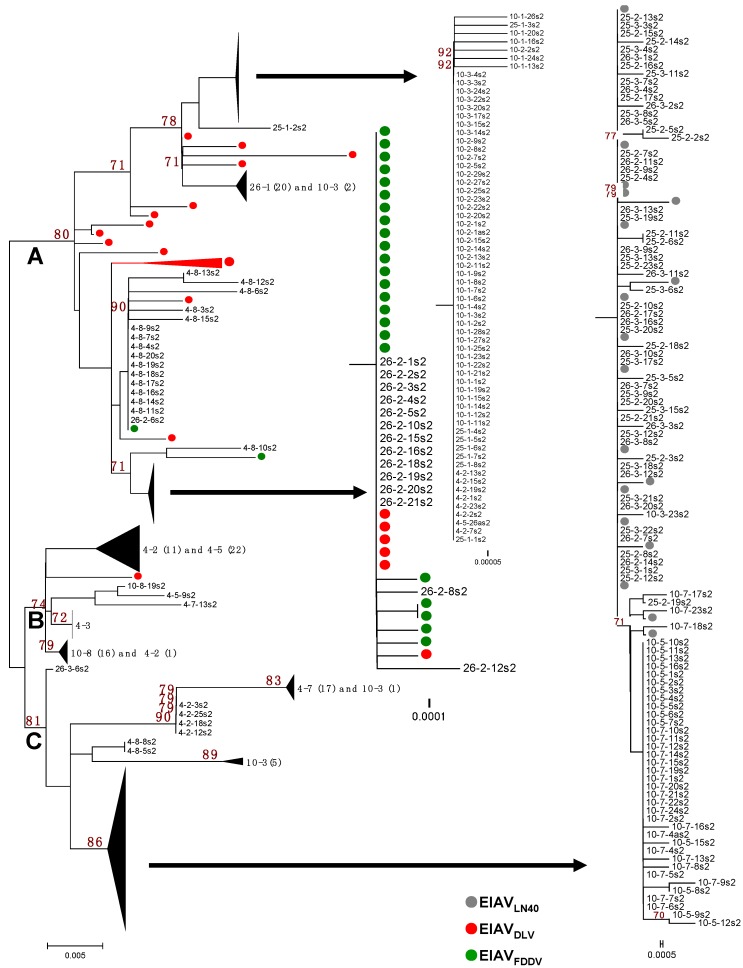
Phylogenetic analysis of the *S2* gene. Sequences include the EIAV_LN40_ stock virus, plasma viral RNA obtained from four EIAV_LN40_-infected horses, the donkey monocyte-derived macrophage (MDM)-adapted vaccine strain EIAV_DLV121_, and the donkey dermal (FDD) cell-attenuated vaccine strain EIAV_FDDV13_. The phylogenetic trees were constructed by the neighbor-joining method, calculated with the Kimura 2 parameter in MEGA software (version 5.0, Center for Evolutionary Functional Genomics Biodesign Institute, Arizona State University, Tempe, AZ, USA, 2011).

The phylogenetic analyses of the *S2* gene sequences of the EIAV_LN40_ clones isolated from the infected horses at different sampling times were performed by comparing the sequences EIAV_LN40_ before inoculation with the sequences of two attenuated vaccines strains, which consisted of 26 clones of EIAV_DLV121_ and 25 clones of EIAV_FDDV13_. The phylogenetic analysis showed that the *S2* sequences were obviously split into three branches, Branches A, B, and C, on the phylogenetic tree (Figure 2). The sequences of EIAV_LN40_ isolated from the horses at different infection stages were identified in different branches that correlated with the period of infection and the clinical symptoms of the host, which were largely determined by the doses of inoculated virus.

Branch A contained all of the clones of samples 4-8, 10-1, 10-2, 25-1, and 26-1 and some of the clones of samples 4-2, 10-3, and 26-2, as well as all of the clones of the EIAV_DLV121_ and EIAV_FDDV13_ vaccine strains (Figure 2). Except for sample 4-8, all of the clones clustered in this branch were isolated from the horses in the early stages of EIAV_LN40_ infection (14 to 28 dpi). In particular, the clones of samples 10-1, 10-2, 10-3, and 25-1 and some of the clones of sample 4-2 were highly homologous, and the following mutations were identified: 22E/K, 39G/E, 41T/I, 51T/I and 55Q/K (Figure 1 and Figure S1). Additionally, the same sub-branch of Branch A included all of the clones of 26-1, which contained an extra 18V/I mutation and did not contain 22E/K. Twelve clones of sample 26-2 had an *S2* gene sequence identical to that of the EIAV vaccine strains (EIAV_DLV121_ and EIAV_FDDV13_, which were attenuated *in vitro*) and were co-located at the same sub-branch of Branch A. The mutations identified were 37K/R, 41T/I, 51T/I and 55Q/K (Figure 1). In addition, the *S2* sequence of a few clones of the vaccine strains clustered with clones of sample 4-8 at the same sub-branch of Branch A. The following mutations were found: 41T/I, 51T/I and 55Q/K (Figure 1). Further analysis revealed that with the exception of 6K/R and 7G/R, all consensus mutations were predominantly observed in sequences clustered in Branch A (Table 3).

**Table 3:** Differences between S2 sequences derived from Branch A and Branch C.

S2 Variation ^a^	No. (%) of Occurrences in:		*p* Value ^b^
	A (*n* = 192)	C (*n* = 144)	
6K/R	25(13)	22(15)	> 0.05
7G/R	0(0)	38(26)	< 0.01
17G/E	8(4)	0(0)	< 0.05
18V/I	22(11)	0(0)	< 0.01
22E/K	64(33)	0(0)	< 0.01
37K/R	72(38)	24(17)	< 0.01
39G/E	93(48)	0(0)	< 0.01
41T/I	162(84)	7(5)	< 0.01
51T/I	164(85)	0(0)	< 0.01
55Q/K	190(99)	1(1)	< 0.01

^a^ Positions are those indicated by letters denote in Figure 1; ^b^ Determined by the chi-square test of the SAS 9.2.

In contrast, Branch C contained all of the clones of the initial EIAV_LN40_ strain; samples 25-2, 25-3, 26-3, 10-5, and 10-7; 5/20 clones of sample 26-2; 17/18 clones of sample 4-7; 4/23 clones of 4-2; and a few clones of samples 10-3 and 4-8 (Figure 2). These *in vivo* isolated clones included most of the viruses isolated at the time points of clinical EIA presentation (body temperature >39 °C and platelet count <100,000 unit/μL) of the horses inoculated with the higher EIAV dose (1 × 10^6^ TCID_50_), including samples 25-2, 25-3, 26-2, and 26-3. The *S2* gene sequences of these viruses were identical to that of the pathogenic EIAV_LN40_ strain (Figure 1). In addition, the samples from horses inoculated with a lower dose of EIAV (1 × 10^5^ TCID_50_) and isolated after the fever peak (sample 10-5) and sample 10-7 were co-located at the same sub-branch with EIAV_LN40_ and had only one mutated site (7G/R) compared with the initial EIAV_LN40_ sequence (Figure 1). Other mutations in clones, including most of 4-7 and a few of 4-2 and 4-8, formed another sub-branch in Branch C; the mutations were predominantly 6K/R and 37K/R (Figure 1). A comparison of the deduced amino acid sequences of the isolated *S2* genes demonstrated that among the ten consensus mutations presented in Table 3, only 7G/R was specifically generated in sequences clustered in the Branch C, and 6K/R showed no distribution trend in Branches A and C. No other consensus mutations were predominantly detected in sequences clustered in Branch C.

In addition to Branches A and C, which contained the *S2* sequences of the attenuated vaccine strains and the pathogenic strain, respectively, some clones clustered and formed a third branch, Branch B. These clones primarily contained sequences from some samples taken from horses inoculated with a low dose of EIAV, *i.e.*, samples 4-3, 4-5 and 10-8, as well as some of the clones of sample 4-2 and one clone of sample 4-7 (Figure 2). The mutation sites of these clones were varied and included 22E/K, 39G/E, and 41T/I; 37K/R, 41T/I, and 55Q/K; or 37K/R and 55Q/K (Figure 1 and Figure S1).

To investigate whether the aforementioned amino acid substitutions in S2 were associated with different stages and statuses of infection, correlations between S2 consensus mutations and the pathogenesis, as well as the phase of infection were analyzed. Sequences of samples 25-2, 25-3, 26-2, 26-3 and 10-5 were grouped as symptomatic, and sequences from the other time points were grouped as asymptomatic. As examined by the Pearsonʼs chi-squared (X^2^) test, the results demonstrated that with the exceptions of 7G/R and 17G/E, eight of the ten primary mutations were significantly correlated with the asymptomatic time points (Table S1). Further, the aforementioned mutation pattern was also significantly correlated with the samples taken during the early phase (earlier than 15 dpi) and the late phase (later than 15 dpi) of infection, when examined by the X^2^ test (Table S2). Combined with the analytic data of the location of mutated sequences in the branches of the phylogenetic tree shown in Figure 2, statistical analyses of the ten consensus mutated residues in S2 revealed that most of these mutations were generated in a specific group of EIAV species and were clustered in Branch A, which emerged in only a particular phase of infection. The quasispecies of pathogenic EIAV_LN40_ with or without these mutations exhibited different capabilities to cause active EIA (Table 3).

## 4. Discussion

In this study, we found that the *S2* genes of pathogenic EIAV_LN40_ strains were highly diverse at different infectious stages, which correlated with the doses of inoculated virus. The higher and lower doses mimicked the acute and chronic EIA, respectively. The divergence of the S2 protein sequence between EIAV isolated *in vivo*, as well as that of EIAV_LN40_ before inoculation, ranged from 0%–14.20%. As many as 14.7% (10 of 68) of the residues were found to be mutated in at least three of the 387 total sequenced clones isolated during 150 days of an *in vivo* inoculation course in four horses. Phylogenetic analysis revealed that these *in vivo* isolated *S2* sequences could be separated into three groups, attenuated-specific Branch A, pathogenic-specific Branch C, and Branch B, which was located between Branches A and C. Interestingly, in all four experimentally infected horses, the original *S2* sequences of the inoculated EIAV_LN40_ were invariably replaced, either completely or partially (partially in horse #4, in which sequences from the first time point were not detectable), by the vaccine-specific sequence (shifted from Branch C to Branch A of the phylogenetic tree). A similar and important observation is that the pathogenic-specific S2, which is identical or very similar to the initial inoculating virus in amino acid sequence, re-emerged after a particular period, either the short period of 15 dpi in horses infected with a high dose of EIAV (1 × 10^6^ TCID_50_) or the long period of approximately 40 dpi to 100 dpi (with the intermediate phase in Branch B) in horses infected with a low dose of the virus (1 × 10^5^ TCID_50_). Statistical analysis revealed that ten primary mutations were significantly different in viruses isolated from time points of active disease or subclinical infection as well as in viruses isolated from the early or late stages of infection (Tables S1 and S2), suggesting an evolutionary tendency of the *S2* gene *in vivo*. Most of these mutations were determined to have resulted from positive selection pressure. These results revealed that *in vivo* selection pressure promotes longitudinal sequence changes of *S2* to allow for adaptation to the host, which in turn alters EIAV pathogenicity, possibly via concurrent effects of mutations in other viral genes. The selective forces that drive these sequence variations and the phenotypic appearance of these viruses isolated from infected hosts will need to be investigated in future studies.

The results in this study showed that the clones from samples 25-2, 25-3, 26-3, 10-5, and 10-7 and 5/20 clones from sample 26-2 are located in the same sub-branch on Branch C, along with the initial EIAV_LN40_ strain. Particularly, the S2 sequences of clones isolated just after febrile episodes, *i.e.*, samples 25-2, 25-3, 26-3 and 26-2 (5/20), were identical to that of the initial EIAV_LN40_ strain, which was previously obtained from the plasma of an EIAV-infected horse during a febrile episode. Sample 26-2, which was obtained from the plasma at the beginning of the first febrile episode, contained both pathogenic-specific and vaccine-specific clones. The pathogenic-specific clones became dominant when detected from sample 26-3 at the time just before death; most of the sequences were identical to those of the initial EIAV_LN40_ strain. These lines of evidence indicate the re-emergence of the pathogenic-specific *S2* sequence, which is correlated with active EIA (Table S1). In addition, although clones from samples 10-5 and 10-7 clustered in Branch C, their sequences differed by one residue (7G/R) from EIAV_LN40_. Although this horse (#10) did not present active EIA, several waves of slight fever (rectal temperature of approximately 39 °C) and minor decreases in the platelet count (below 100,000/µL) occurred before sample 10-5 was collected (Figure 1). These results suggest that the aforementioned longitudinal S2 sequence alteration is associated with disease development (Table 3, Tables S1 and S2). Notably, the virus in horses #4 and #10 did not re-emerge with the EIAV_LN40_ sequence, as was observed in horses #25 and #26, but eventually did evolve into Branch B or mixed A/C and B/C (Figure S1). We presume that certain levels of immunity were induced after the initial infection. If the horses do not die of acute EIA, then the immune suppression drives EIAV to further evolve into a less pathogenic and/or latent status. This hypothesis is supported by previous reports indicating that horses that clinically recovered from chronic EIA acquired resistance to subsequent EIAV infection [17].

At the early phase of infection, we observed the appearance of clones containing *S2* sequences clustered with the sequence of vaccine strains, which implies the selection of vaccine-like viral species in the quasispecies pool of EIAV, as well as the re-emergence of clones containing *S2* sequences clustered with the sequence of EIAV_LN40_ strain at the late infection phase; this pattern of sequence clustering is correlated with typical EIA clinical symptoms. This tendency for *in vivo* evolution was observed in the *S2* gene of all four horses examined in this study as well as in the previously examined *gp90* gene [13]. This feature of EIAV *in vivo* evolution is consistent with the trophic change of HIV-1 in different infection phases and the re-boost of latent strains in clinically cured patients [18]. A general pattern might exist for the adaption of pathogenic EIAV_LN40_ in the host. Our earlier studies on cultivated primary target cells of EIAV revealed that the pathogenic and vaccine strains induced different panels of cytokines at different stages of infection [19,20]. A particular phenotype pattern, including the viral structure and function controlled by *S2* and *gp90*, is considered beneficial in a specific infection phase to successfully establish infection. Studies have shown that EIAV with wild-type *S2* induced significantly higher levels of inflammatory cytokines and chemokines than the *S2*-deficient control in eMDMs. It has been proposed that the enhanced cytokines created a suitable intracellular environment for viral infection and replication and affected the disease development [21]. The clinical signs (febrile responses, thrombocytopenia and anemia) associated with acute EIA are mediated by pro-inflammatory cytokines, such as TNFα, IL-6, and TGFβ [22]. A number of studies on *lentiviruses*, including HIV-1, SIV and feline immunodeficiency virus (FIV), have confirmed that the expression of a variety of cytokines could affect viral replication and disease progression.

The emergence of quasispecies containing the mutated *S2* gene could be considered the result of the generation of new mutations in predominant viral species, the selection of existing underrepresented species, or both. The editing activities of APOBECs and ADAR 1 are major known selection pressures that stimulate G to A and A to G mutations in retroviruses and double-stranded RNA viruses [23,24]. An altered environment could boost the replication of certain existing underrepresented viral species. In a previous study, using the single-genome amplification (SGA) method, we identified 41 unique *gp90* V3–V5 sequences from 73 clones of the EIAV vaccine strain EIAV_FDDV13_, among which contained nine sequences with a 3-nucleotide insert that was previously identified only in the sequences of pathogenic strains [25]. Studies on HIV-1 found that a single transmitted founder (TF) virus, which possesses specific phenotypic properties compared with chronic control viruses, is sufficient to establish a new infection [26]. Clones containing the vaccine-specific *S2* gene isolated from EIAV_LN40_-infected horses could be selected from existing viral species. If this finding was true, an extensive sequence analysis of specific quasispecies of a *lentivirus* strain would facilitate antigen design for vaccine development.

## Supplementary Files

Supplementary File 1
