# Supplementary material for: A Unique Evolution of the S2 Gene of Equine Infectious Anemia Virus in Hosts Correlated with Particular Infection Statuses
Source: Viruses. 2014 Nov 10;6(11):4265–79. doi: 10.3390/v6114265 (PMC4246221; doi:10.3390/v6114265)
Supplement: Supplementary File 1 [file viruses-06-04265-s001.pdf]

# Supplementary Materials

## A Unique Evolution of the *S2* Gene of Equine Infectious Anemia Virus in Hosts Correlated with Particular Infection Statuses

**Xue-Feng Wang, Shuai Wang, Qiang Liu, Yue-Zhi Lin, Cheng Du, Yan-Dong Tang, Lei Na, Xiaojun Wang and Jian-Hua Zhou**

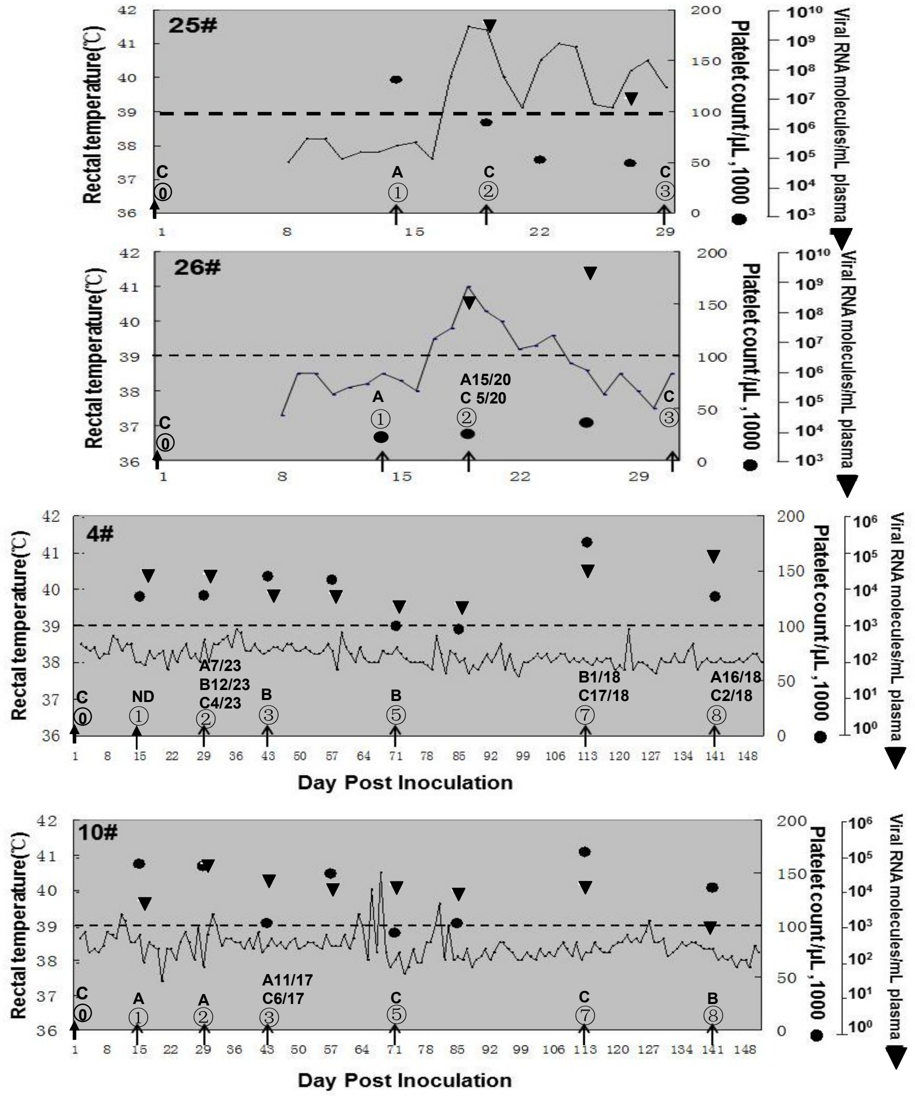


**Figure S1.** The clinical profile of the horses experimentally infected with $EIAV_{LN40}$. The rectal temperature (solid lines, the left *Y*-axis) and platelet count (spots, the first right *Y*-axis) and quantitation of the virus load (arrowheads, the second right *Y*-axis) were monitored at the indicated time points after infection as standard measures of disease. "0" indicates the inoculated point, "1", "2", "3"…indicates the sampled point. The letter A, B and C represent the corresponding branch of the phylogenetic tree, respectively. ND indicates not detectable. The febrile episodes were defined by rectal temperatures above 39 °C, in conjunction with a reduction in the platelet counts to below 100,000/μL of whole blood and other characteristic clinical symptoms.

**Table S1.** Differences between *S2* sequences derived from samples taken from symptomatic and asymptomatic time points.

| S2 Variation [a] | No. (%) of Occurrences in Time Points of | | *p* value [b] |
|---|---|---|---|
| | Symptomatic ($n$ = 106) | Asymptomatic ($n$ = 280) | |
| 6K/R | 0(0) | 58(21) | <0.01 |
| 7G/R | 15(14) | 23(8) | $p > 0.05$ |
| 17G/E | 0(0) | 8(3) | $p > 0.05$ |
| 18V/I | 0(0) | 22(8) | <0.01 |
| 22E/K | 0(0) | 65(23) | <0.01 |
| 37K/R | 14(13) | 132(47) | <0.01 |
| 39G/E | 0(0) | 93(33) | <0.01 |
| 41T/I | 15(14) | 175(63) | <0.01 |
| 51T/I | 15(14) | 150(54) | <0.01 |
| 55Q/K | 15(14) | 225(80) | <0.01 |

[a] Positions are those indicated by red denote in Figure 1; [b] Determined by the chi-square test of the SAS 9.2.

**Table S2**. Differences between S2 sequences derived from the early phase or late phase of EIAV infection.

| S2 Variation [a] | No. (%) of Occurrences in: | | *p* value [b] |
|---|---|---|---|
| | Early phase ($n$ = 52) | Late phase ($n$ = 283) | |
| 6K/R | 0(0) | 56(20) | <0.01 |
| 7G/R | 0(0) | 38(13) | <0.01 |
| 17G/E | 0(0) | 0(0) | - |
| 18V/I | 32(62) | 2(1) | <0.01 |
| 22E/K | 31(60) | 34(12) | <0.01 |
| 37K/R | 0(0) | 111(39) | <0.01 |
| 39G/E | 50(96) | 35(12) | <0.01 |
| 41T/I | 51(98) | 94(33) | <0.01 |
| 51T/I | 50(96) | 69(24) | <0.01 |
| 55Q/K | 50(96) | 140(49) | <0.01 |

[a] Positions are those indicated by red denote in Figure 1; [b] Determined by the chi-square test of the SAS 9.2.
